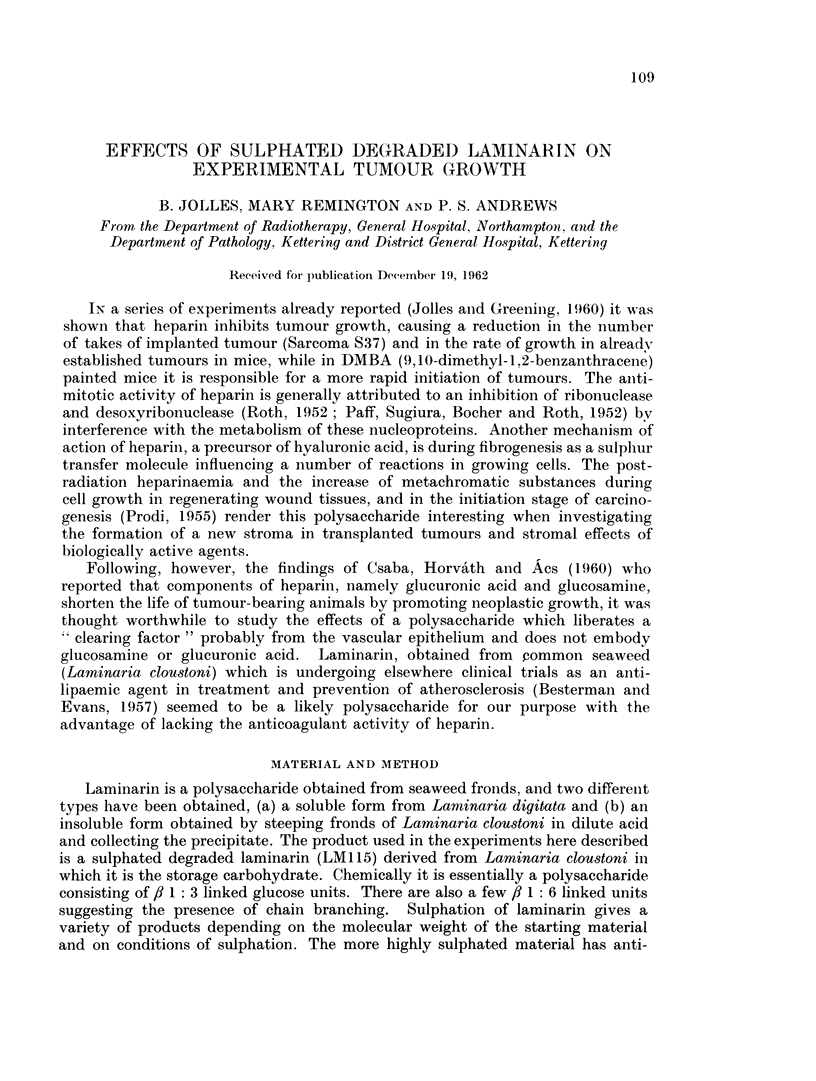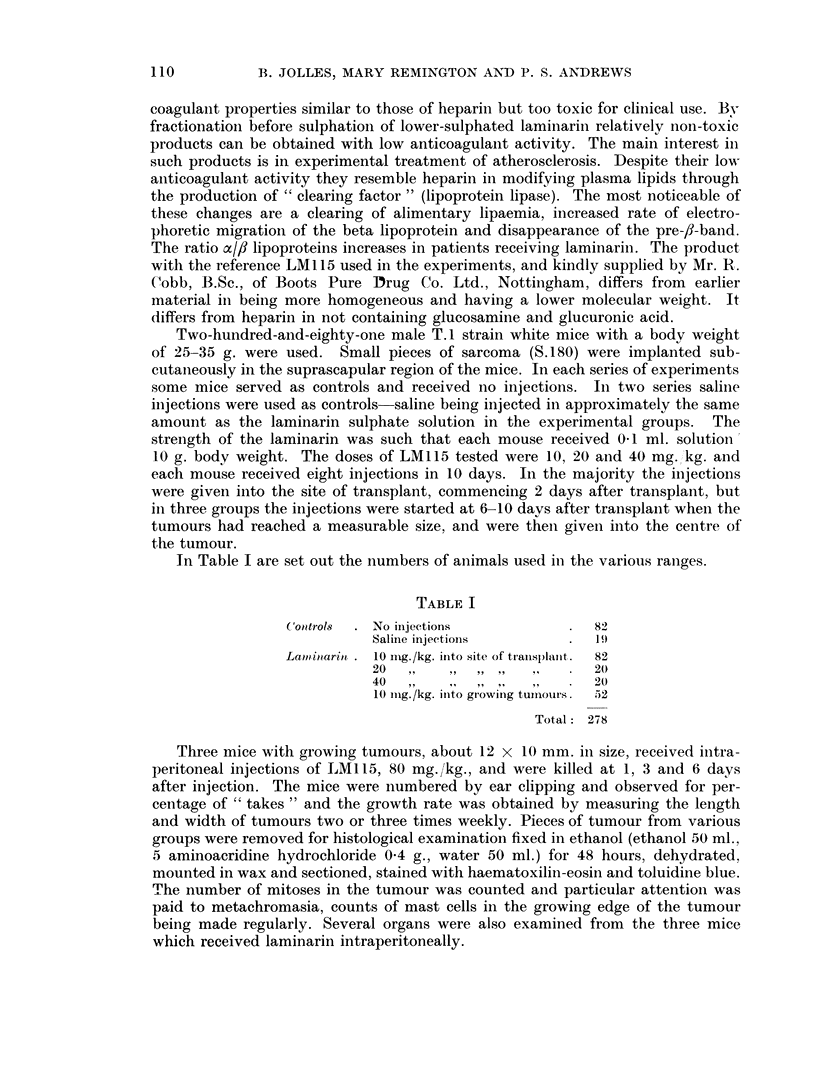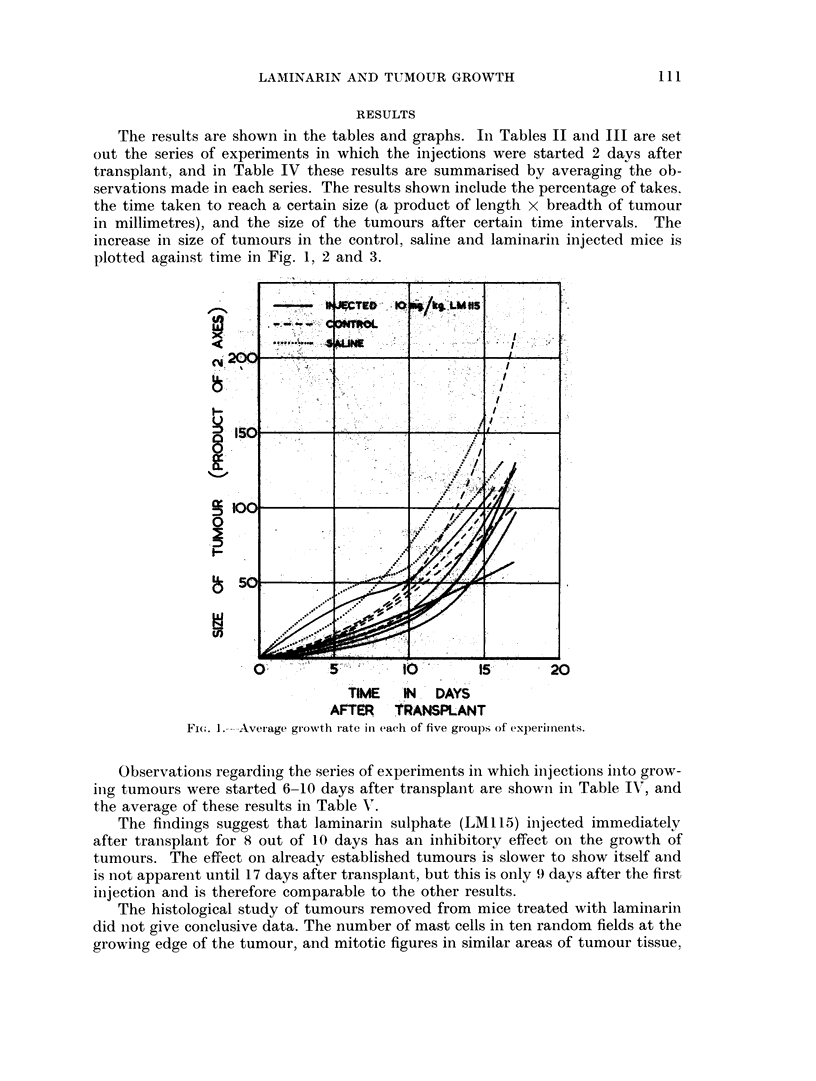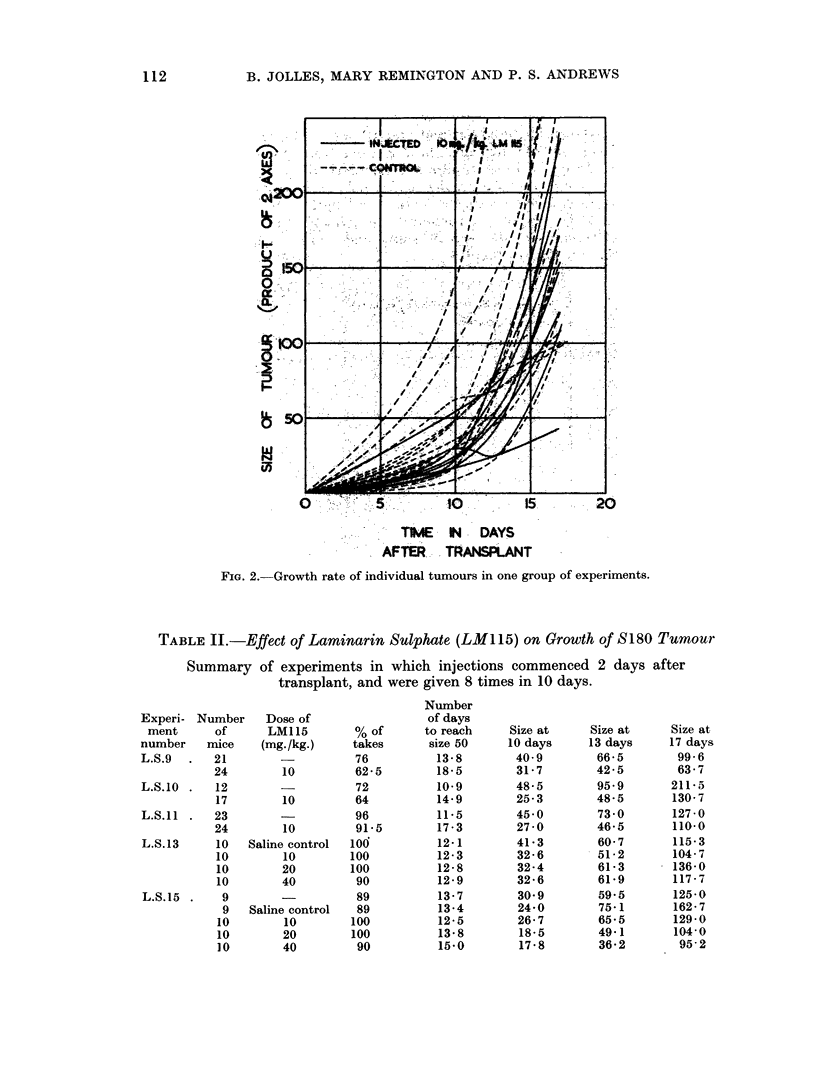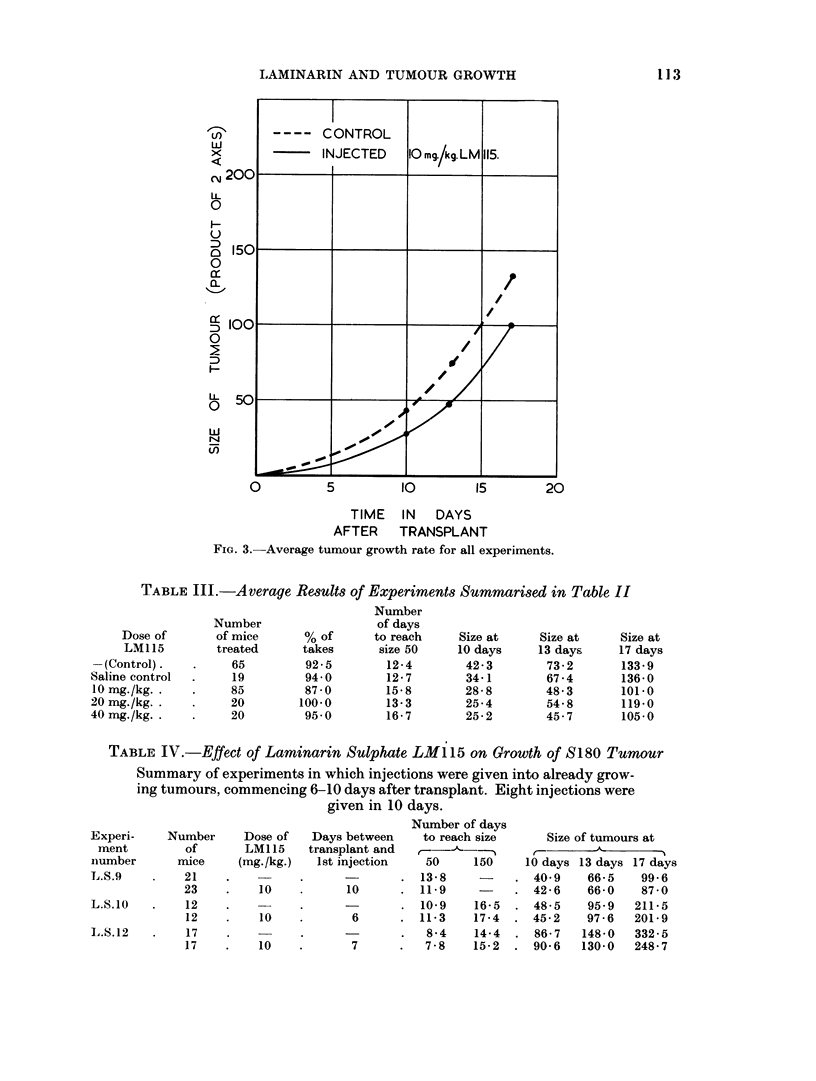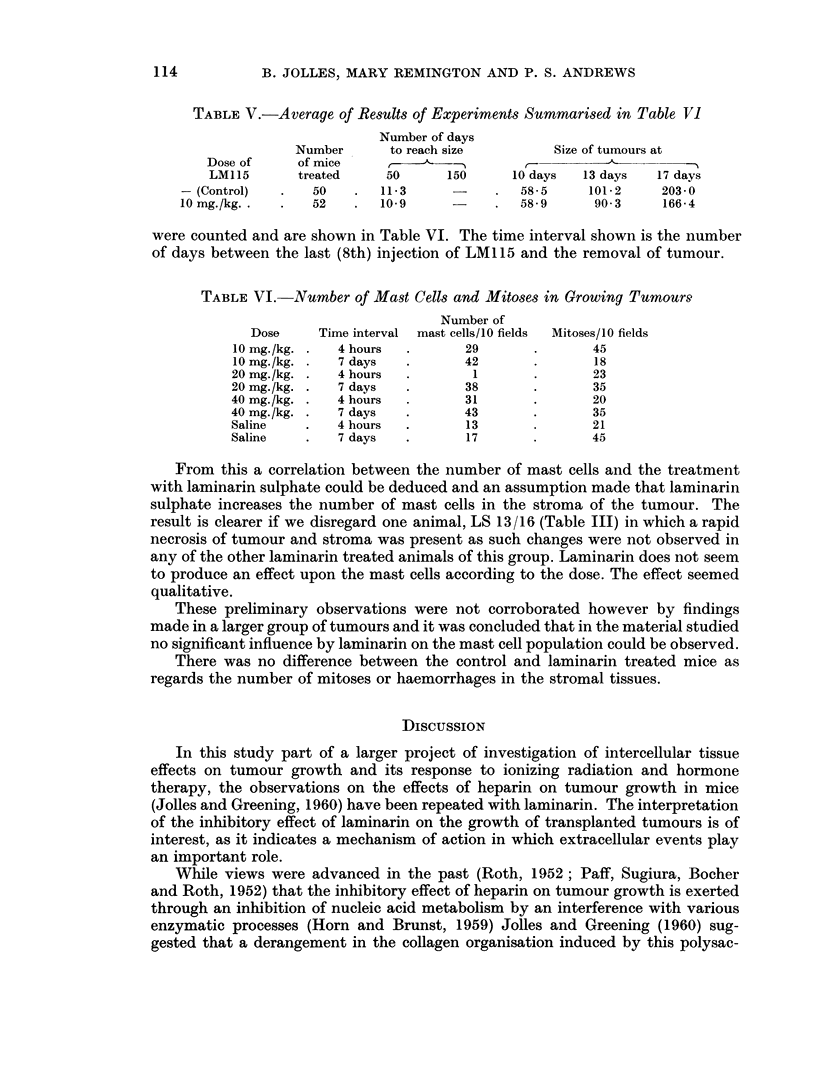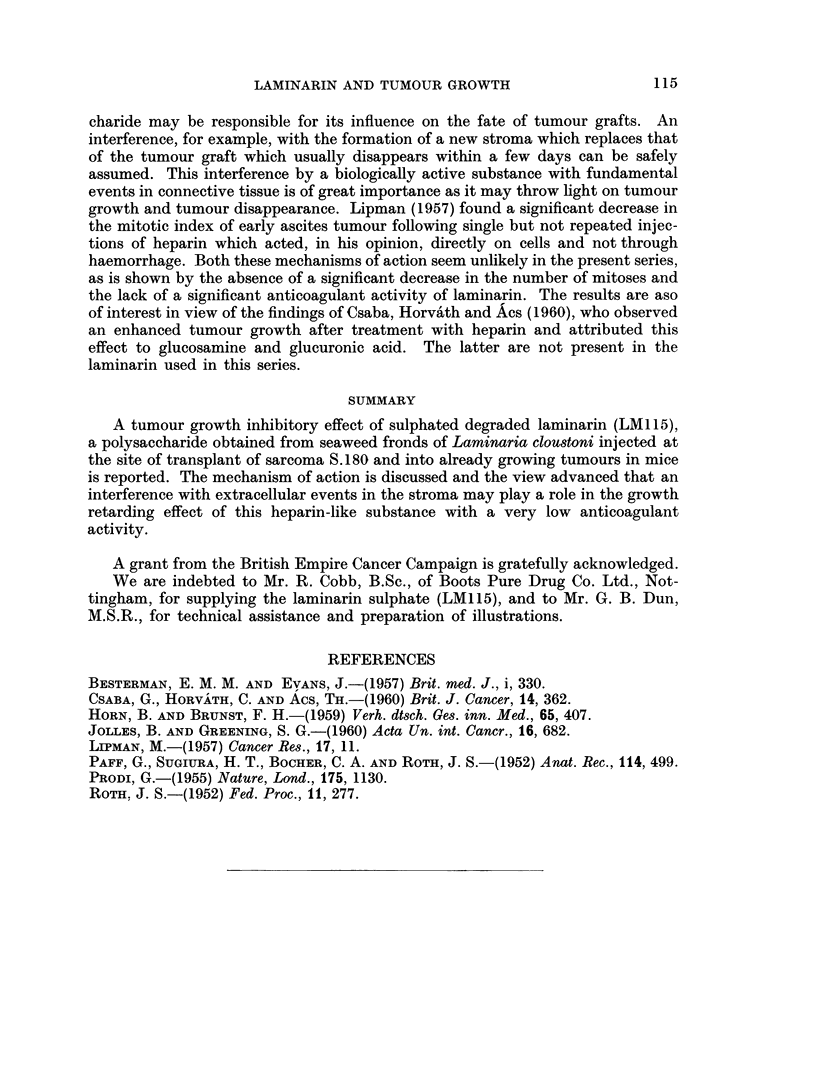# Effects of Sulphated Degraded Laminarin on Experimental Tumour Growth

**DOI:** 10.1038/bjc.1963.16

**Published:** 1963-03

**Authors:** B. Jolles, Mary Remington, P. S. Andrews


### EFFECTS OF SULPHATED DEGRADED LAMINARIN ON EXPERIMENTAL TUMOUR GROWTH

B. JOLLES, MARY REMINGTON AND P. S. ANDREWS

*From the Department of Radiotherapy, General Hospital, Northampton, and the Department of Pathology, Kettering and District General Hospital, Kettering*

Received for publication December 19, 1962

In a series of experiments already reported (Jolles and Greening, 1960) it was shown that heparin inhibits tumour growth, causing a reduction in the number of takes of implanted tumour (Sarcoma S37) and in the rate of growth in already established tumours in mice, while in DMBA (9,10-dimethyl-1,2-benzanthracene) painted mice it is responsible for a more rapid initiation of tumours. The anti-mitotic activity of heparin is generally attributed to an inhibition of ribonuclease and desoxyribonuclease (Roth, 1952 ; Paff, Sugiura, Bocher and Roth, 1952) by interference with the metabolism of these nucleoproteins. Another mechanism of action of heparin, a precursor of hyaluronic acid, is during fibrogenesis as a sulphur transfer molecule influencing a number of reactions in growing cells. The post-radiation heparinaemia and the increase of metachromatic substances during cell growth in regenerating wound tissues, and in the initiation stage of carcinogenesis (Prodi, 1955) render this polysaccharide interesting when investigating the formation of a new stroma in transplanted tumours and stromal effects of biologically active agents.

Following, however, the findings of Csaba, Horváth and Ács (1960) who reported that components of heparin, namely glucuronic acid and glucosamine, shorten the life of tumour-bearing animals by promoting neoplastic growth, it was thought worthwhile to study the effects of a polysaccharide which liberates a " clearing factor " probably from the vascular epithelium and does not embody glucosamine or glucuronic acid. Laminarin, obtained from common seaweed (*Laminaria cloustoni*) which is undergoing elsewhere clinical trials as an anti-lipaemic agent in treatment and prevention of atherosclerosis (Besterman and Evans, 1957) seemed to be a likely polysaccharide for our purpose with the advantage of lacking the anticoagulant activity of heparin.

### MATERIAL AND METHOD

Laminarin is a polysaccharide obtained from seaweed fronds, and two different types have been obtained, (a) a soluble form from *Laminaria digitata* and (b) an insoluble form obtained by steeping fronds of *Laminaria cloustoni* in dilute acid and collecting the precipitate. The product used in the experiments here described is a sulphated degraded laminarin (LM115) derived from *Laminaria cloustoni* in which it is the storage carbohydrate. Chemically it is essentially a polysaccharide consisting of $\beta$ 1 : 3 linked glucose units. There are also a few $\beta$ 1 : 6 linked units suggesting the presence of chain branching. Sulphation of laminarin gives a variety of products depending on the molecular weight of the starting material and on conditions of sulphation. The more highly sulphated material has anticoagulant properties similar to those of heparin but too toxic for clinical use. By fractionation before sulphation of lower-sulphated laminarin relatively non-toxic products can be obtained with low anticoagulant activity. The main interest in such products is in experimental treatment of atherosclerosis. Despite their low anticoagulant activity they resemble heparin in modifying plasma lipids through the production of " clearing factor " (lipoprotein lipase). The most noticeable of these changes are a clearing of alimentary lipaemia, increased rate of electrophoretic migration of the beta lipoprotein and disappearance of the pre-$\beta$-band. The ratio $\alpha/\beta$ lipoproteins increases in patients receiving laminarin. The product with the reference LM115 used in the experiments, and kindly supplied by Mr. R. Cobb, B.Sc., of Boots Pure Drug Co. Ltd., Nottingham, differs from earlier material in being more homogeneous and having a lower molecular weight. It differs from heparin in not containing glucosamine and glucuronic acid.

Two-hundred-and-eighty-one male T.1 strain white mice with a body weight of 25–35 g. were used. Small pieces of sarcoma (S.180) were implanted subcutaneously in the suprascapular region of the mice. In each series of experiments some mice served as controls and received no injections. In two series saline injections were used as controls—saline being injected in approximately the same amount as the laminarin sulphate solution in the experimental groups. The strength of the laminarin was such that each mouse received 0·1 ml. solution/10 g. body weight. The doses of LM115 tested were 10, 20 and 40 mg./kg. and each mouse received eight injections in 10 days. In the majority the injections were given into the site of transplant, commencing 2 days after transplant, but in three groups the injections were started at 6–10 days after transplant when the tumours had reached a measurable size, and were then given into the centre of the tumour.

In Table I are set out the numbers of animals used in the various ranges.

TABLE I

| | | |
|---|---|---|
| *Controls* . | No injections . | 82 |
| | Saline injections . | 19 |
| *Laminarin* . | 10 mg./kg. into site of transplant. | 82 |
| | 20 ,, ,, ,, ,, ,, . | 20 |
| | 40 ,, ,, ,, ,, ,, . | 20 |
| | 10 mg./kg. into growing tumours. | 52 |
| | Total : | 278 |

Three mice with growing tumours, about 12 × 10 mm. in size, received intraperitoneal injections of LM115, 80 mg./kg., and were killed at 1, 3 and 6 days after injection. The mice were numbered by ear clipping and observed for percentage of " takes " and the growth rate was obtained by measuring the length and width of tumours two or three times weekly. Pieces of tumour from various groups were removed for histological examination fixed in ethanol (ethanol 50 ml., 5 aminoacridine hydrochloride 0·4 g., water 50 ml.) for 48 hours, dehydrated, mounted in wax and sectioned, stained with haematoxilin-eosin and toluidine blue. The number of mitoses in the tumour was counted and particular attention was paid to metachromasia, counts of mast cells in the growing edge of the tumour being made regularly. Several organs were also examined from the three mice which received laminarin intraperitoneally.

RESULTS

The results are shown in the tables and graphs. In Tables II and III are set out the series of experiments in which the injections were started 2 days after transplant, and in Table IV these results are summarised by averaging the observations made in each series. The results shown include the percentage of takes, the time taken to reach a certain size (a product of length × breadth of tumour in millimetres), and the size of the tumours after certain time intervals. The increase in size of tumours in the control, saline and laminarin injected mice is plotted against time in Fig. 1, 2 and 3.

FIG. 1.—Average growth rate in each of five groups of experiments.

Observations regarding the series of experiments in which injections into growing tumours were started 6–10 days after transplant are shown in Table IV, and the average of these results in Table V.

The findings suggest that laminarin sulphate (LM115) injected immediately after transplant for 8 out of 10 days has an inhibitory effect on the growth of tumours. The effect on already established tumours is slower to show itself and is not apparent until 17 days after transplant, but this is only 9 days after the first injection and is therefore comparable to the other results.

The histological study of tumours removed from mice treated with laminarin did not give conclusive data. The number of mast cells in ten random fields at the growing edge of the tumour, and mitotic figures in similar areas of tumour tissue,

FIG. 2.—Growth rate of individual tumours in one group of experiments.

TABLE II.—*Effect of Laminarin Sulphate* (*LM*115) *on Growth of S*180 *Tumour*

Summary of experiments in which injections commenced 2 days after transplant, and were given 8 times in 10 days.

| Experiment number | Number of mice | Dose of LM115 (mg./kg.) | % of takes | Number of days to reach size 50 | Size at 10 days | Size at 13 days | Size at 17 days |
|---|---|---|---|---|---|---|---|
| L.S.9 . | 21 | — | 76 | 13·8 | 40·9 | 66·5 | 99·6 |
| | 24 | 10 | 62·5 | 18·5 | 31·7 | 42·5 | 63·7 |
| L.S.10 . | 12 | — | 72 | 10·9 | 48·5 | 95·9 | 211·5 |
| | 17 | 10 | 64 | 14·9 | 25·3 | 48·5 | 130·7 |
| L.S.11 . | 23 | — | 96 | 11·5 | 45·0 | 73·0 | 127·0 |
| | 24 | 10 | 91·5 | 17·3 | 27·0 | 46·5 | 110·0 |
| L.S.13 | 10 | Saline control | 100 | 12·1 | 41·3 | 60·7 | 115·3 |
| | 10 | 10 | 100 | 12·3 | 32·6 | 51·2 | 104·7 |
| | 10 | 20 | 100 | 12·8 | 32·4 | 61·3 | 136·0 |
| | 10 | 40 | 90 | 12·9 | 32·6 | 61·9 | 117·7 |
| L.S.15 . | 9 | — | 89 | 13·7 | 30·9 | 59·5 | 125·0 |
| | 9 | Saline control | 89 | 13·4 | 24·0 | 75·1 | 162·7 |
| | 10 | 10 | 100 | 12·5 | 26·7 | 65·5 | 129·0 |
| | 10 | 20 | 100 | 13·8 | 18·5 | 49·1 | 104·0 |
| | 10 | 40 | 90 | 15·0 | 17·8 | 36·2 | 95·2 |

FIG. 3.—Average tumour growth rate for all experiments.

TABLE III.—*Average Results of Experiments Summarised in Table II*

| Dose of LM115 | Number of mice treated | % of takes | Number of days to reach size 50 | Size at 10 days | Size at 13 days | Size at 17 days |
|---|---|---|---|---|---|---|
| —(Control) . | 65 | 92·5 | 12·4 | 42·3 | 73·2 | 133·9 |
| Saline control | 19 | 94·0 | 12·7 | 34·1 | 67·4 | 136·0 |
| 10 mg./kg. . | 85 | 87·0 | 15·8 | 28·8 | 48·3 | 101·0 |
| 20 mg./kg. . | 20 | 100·0 | 13·3 | 25·4 | 54·8 | 119·0 |
| 40 mg./kg. . | 20 | 95·0 | 16·7 | 25·2 | 45·7 | 105·0 |

TABLE IV.—*Effect of Laminarin Sulphate LM*115 *on Growth of S*180 *Tumour*

Summary of experiments in which injections were given into already growing tumours, commencing 6–10 days after transplant. Eight injections were given in 10 days.

| Experiment number | Number of mice | Dose of LM115 (mg./kg.) | Days between transplant and 1st injection | Number of days to reach size | | Size of tumours at | | |
|---|---|---|---|---|---|---|---|---|
| | | | | 50 | 150 | 10 days | 13 days | 17 days |
| L.S.9 | 21 | — | — | 13·8 | — | 40·9 | 66·5 | 99·6 |
| | 23 | 10 | 10 | 11·9 | — | 42·6 | 66·0 | 87·0 |
| L.S.10 | 12 | — | — | 10·9 | 16·5 | 48·5 | 95·9 | 211·5 |
| | 12 | 10 | 6 | 11·3 | 17·4 | 45·2 | 97·6 | 201·9 |
| L.S.12 | 17 | — | — | 8·4 | 14·4 | 86·7 | 148·0 | 332·5 |
| | 17 | 10 | 7 | 7·8 | 15·2 | 90·6 | 130·0 | 248·7 |

TABLE V.—*Average of Results of Experiments Summarised in Table VI*

| Dose of LM115 | Number of mice treated | Number of days to reach size | | Size of tumours at | | |
|---|---|---|---|---|---|---|
| | | 50 | 150 | 10 days | 13 days | 17 days |
| — (Control) | 50 | 11·3 | — | 58·5 | 101·2 | 203·0 |
| 10 mg./kg. | 52 | 10·9 | — | 58·9 | 90·3 | 166·4 |

were counted and are shown in Table VI. The time interval shown is the number of days between the last (8th) injection of LM115 and the removal of tumour.

TABLE VI.—*Number of Mast Cells and Mitoses in Growing Tumours*

| Dose | Time interval | Number of mast cells/10 fields | Mitoses/10 fields |
|---|---|---|---|
| 10 mg./kg. | 4 hours | 29 | 45 |
| 10 mg./kg. | 7 days | 42 | 18 |
| 20 mg./kg. | 4 hours | 1 | 23 |
| 20 mg./kg. | 7 days | 38 | 35 |
| 40 mg./kg. | 4 hours | 31 | 20 |
| 40 mg./kg. | 7 days | 43 | 35 |
| Saline | 4 hours | 13 | 21 |
| Saline | 7 days | 17 | 45 |

From this a correlation between the number of mast cells and the treatment with laminarin sulphate could be deduced and an assumption made that laminarin sulphate increases the number of mast cells in the stroma of the tumour. The result is clearer if we disregard one animal, LS 13/16 (Table III) in which a rapid necrosis of tumour and stroma was present as such changes were not observed in any of the other laminarin treated animals of this group. Laminarin does not seem to produce an effect upon the mast cells according to the dose. The effect seemed qualitative.

These preliminary observations were not corroborated however by findings made in a larger group of tumours and it was concluded that in the material studied no significant influence by laminarin on the mast cell population could be observed.

There was no difference between the control and laminarin treated mice as regards the number of mitoses or haemorrhages in the stromal tissues.

### DISCUSSION

In this study part of a larger project of investigation of intercellular tissue effects on tumour growth and its response to ionizing radiation and hormone therapy, the observations on the effects of heparin on tumour growth in mice (Jolles and Greening, 1960) have been repeated with laminarin. The interpretation of the inhibitory effect of laminarin on the growth of transplanted tumours is of interest, as it indicates a mechanism of action in which extracellular events play an important role.

While views were advanced in the past (Roth, 1952 ; Paff, Sugiura, Bocher and Roth, 1952) that the inhibitory effect of heparin on tumour growth is exerted through an inhibition of nucleic acid metabolism by an interference with various enzymatic processes (Horn and Brunst, 1959) Jolles and Greening (1960) suggested that a derangement in the collagen organisation induced by this polysaccharide may be responsible for its influence on the fate of tumour grafts. An interference, for example, with the formation of a new stroma which replaces that of the tumour graft which usually disappears within a few days can be safely assumed. This interference by a biologically active substance with fundamental events in connective tissue is of great importance as it may throw light on tumour growth and tumour disappearance. Lipman (1957) found a significant decrease in the mitotic index of early ascites tumour following single but not repeated injections of heparin which acted, in his opinion, directly on cells and not through haemorrhage. Both these mechanisms of action seem unlikely in the present series, as is shown by the absence of a significant decrease in the number of mitoses and the lack of a significant anticoagulant activity of laminarin. The results are aso of interest in view of the findings of Csaba, Horváth and Ács (1960), who observed an enhanced tumour growth after treatment with heparin and attributed this effect to glucosamine and glucuronic acid. The latter are not present in the laminarin used in this series.

SUMMARY

A tumour growth inhibitory effect of sulphated degraded laminarin (LM115), a polysaccharide obtained from seaweed fronds of *Laminaria cloustoni* injected at the site of transplant of sarcoma S.180 and into already growing tumours in mice is reported. The mechanism of action is discussed and the view advanced that an interference with extracellular events in the stroma may play a role in the growth retarding effect of this heparin-like substance with a very low anticoagulant activity.

A grant from the British Empire Cancer Campaign is gratefully acknowledged.

We are indebted to Mr. R. Cobb, B.Sc., of Boots Pure Drug Co. Ltd., Nottingham, for supplying the laminarin sulphate (LM115), and to Mr. G. B. Dun, M.S.R., for technical assistance and preparation of illustrations.

### REFERENCES

BESTERMAN, E. M. M. AND EVANS, J.—(1957) *Brit. med. J.*, i, 330.
CSABA, G., HORVÁTH, C. AND ÁCS, TH.—(1960) *Brit. J. Cancer*, **14**, 362.
HORN, B. AND BRUNST, F. H.—(1959) *Verh. dtsch. Ges. inn. Med.*, **65**, 407.
JOLLES, B. AND GREENING, S. G.—(1960) *Acta Un. int. Cancr.*, **16**, 682.
LIPMAN, M.—(1957) *Cancer Res.*, **17**, 11.
PAFF, G., SUGIURA, H. T., BOCHER, C. A. AND ROTH, J. S.—(1952) *Anat. Rec.*, **114**, 499.
PRODI, G.—(1955) *Nature, Lond.*, **175**, 1130.
ROTH, J. S.—(1952) *Fed. Proc.*, **11**, 277.